# Supplementary material for: First Descriptive Analysis of the Faecal Microbiota of Wild and Anthropized Barbary Macaques (Macaca sylvanus) in the Region of Bejaia, Northeast Algeria
Source: Biology (Basel). 2022 Jan 25;11(2):187. doi: 10.3390/biology11020187 (PMC8869477; doi:10.3390/biology11020187)
Supplement: Supplementary file 1 [file biology-11-00187-s001.zip › biology-1490237-supplementary.pdf]

First Descriptive Analysis of the Faecal Microbiota of Wild and Anthropized Barbary Macaques (*Macaca sylvanus*) in the Region of Bejaia, Northeast Algeria

Mourad Boumenir [1,2], Jean-Luc Hornick [1,2], Bernard Taminiau [2,3], Georges Daube [2,3], Fany Brotcorne [4], Mokrane Iguer-Ouada [5] and Nassim Moula [2,6,7,*]

[1] Department of Veterinary Management of Animal Resources, Faculty of Veterinary Medicine, University of Liege, 4000 Liege, Belgium; Mourad.Boumenir@doct.uliege.be (M.B.); jlhornick@uliege.be (J.-L.H.)

[2] Fundamental and Applied Research for Animal and Health (FARAH), University of Liege, 4000 Liege, Belgium; bernard.taminiau@uliege.be (B.T.); Georges.Daube@uliege.be (G.D.)

[3] Food Microbiology, Department of Food Sciences, Faculty of Veterinary Medicine, University of Liege, 4000 Liege, Belgium

[4] Behaviour Biology Lab, Research Unit SPHERES, Department of Biology, Ecology, and Evolution, Faculty of Sciences, University of Liège, 4000 Liege, Belgium; fbrotcorne@uliege.be

[5] Associated Laboratory in Marine Ecosystems and Aquaculture, Department of Biological Sciences of the Environment, Faculty of Nature and Life Sciences, University of Bejaia, 06000 Bejaia, Algeria; imokrane@gmail.com

[6] GIGA Animal Facilities, Liege University, 4000 Liege, Belgium

[7] Department of Animal Production, Faculty of Veterinary Medicine, Liege University, 4000 Liege, Belgium

* Correspondence: Nassim.Moula@uliege.be; Tel.: +32-4-366-4124

Table S1: A measure of sampling effort for each sample using Good's coverage estimator (rarefied table at 10,000 sequences per sample).

Samples label	Good's coverage (%)
TPG 01	99.79%
TPG 02	99.70%
TPG 03	99.71%
TPG 04	99.82%
TPG 05	99.82%
TPG 06	99.78%
WFG 01	99.83%
WFG 02	99.85%
WFG 03	99.69%
WFG 04	99.75%
WFG 05	99.74%
WFG 06	99.77%

Table S2: The relative abundance and the average proportions of the bacterial phyla of Barbary macaque fecal microbiota in both TPG and WFG.

Phyla	Relative abundance (%)												Average proportions (%)	SD
	TPG 1	TPG 2	TPG 3	TPG 4	TPG 5	TPG 6	WFG 1	WFG 2	WFG 3	WFG 4	WFG 5	WFG 6		
**Firmicutes**	91.263	90.384	93.744	97.857	99.036	99.009	91.343	92.396	91.504	97.299	91.460	98.402	94.475	3.514
**Bacteroidetes**	0.423	1.045	1.824	1.157	0.311	0.344	5.309	5.288	7.115	1.091	7.817	0.131	2.655	2.871
**Verrucomicrobia**	4.560	4.974	2.935	0.000	0.050	0.010	0.081	0.040	0.053	0.000	0.000	0.303	1.084	1.910
**Tenericutes**	0.362	0.261	0.703	0.161	0.151	0.273	1.901	0.413	0.171	0.023	0.100	0.607	0.427	0.506
**Actinobacteria**	0.755	0.533	0.214	0.191	0.120	0.071	0.961	1.068	0.171	0.191	0.271	0.081	0.386	0.354
**Spirochaetes**	1.892	2.120	0.010	0.000	0.020	0.000	0.061	0.020	0.000	0.000	0.040	0.040	0.350	0.775
**Proteobacteria**	0.091	0.151	0.153	0.443	0.151	0.152	0.202	0.645	0.396	0.360	0.100	0.131	0.248	0.173
**Bacteria_ph**	0.030	0.060	0.316	0.040	0.060	0.101	0.051	0.091	0.439	0.878	0.030	0.081	0.181	0.253
**Cyanobacteria**	0.030	0.030	0.061	0.141	0.070	0.010	0.091	0.030	0.096	0.079	0.181	0.192	0.084	0.060
**Epsilonbacteraeota**	0.413	0.221	0.031	0.000	0.000	0.000	0.000	0.000	0.011	0.000	0.000	0.000	0.056	0.129
**WPS-2**	0.171	0.181	0.010	0.000	0.000	0.000	0.000	0.000	0.000	0.000	0.000	0.000	0.030	0.068
**Fusobacteria**	0.000	0.000	0.000	0.000	0.010	0.000	0.000	0.000	0.021	0.079	0.000	0.010	0.010	0.023
**Patescibacteria**	0.010	0.000	0.000	0.000	0.020	0.030	0.000	0.000	0.011	0.000	0.000	0.020	0.008	0.011
**Lentisphaerae**	0.000	0.010	0.000	0.000	0.000	0.000	0.000	0.010	0.011	0.000	0.000	0.000	0.003	0.005
**Chloroflexi**	0.000	0.020	0.000	0.000	0.000	0.000	0.000	0.000	0.000	0.000	0.000	0.000	0.002	0.006
**Deinococcus-Thermus**	0.000	0.000	0.000	0.010	0.000	0.000	0.000	0.000	0.000	0.000	0.000	0.000	0.001	0.003
**Gemmatimonadetes**	0.000	0.010	0.000	0.000	0.000	0.000	0.000	0.000	0.000	0.000	0.000	0.000	0.001	0.003

Table S3: The average proportions of Barbary macaque fecal microbiota at phylum level between the TP and WF groups.

Phylum	TPG		WFG	
	Average proportions (%)	S.D (%)	Average proportions (%)	S.D (%)
**Firmicutes**	94.84135765	71.79498625	93.2697723	69.08536031
**Bacteroidetes**	**0.826921863**	0.986793702	**4.297393312**	5.468066568
**Tenericutes**	0.318547131	0.203765701	0.52564215	0.724610482
**Actinobacteria**	0.216688215	0.250110694	0.428547018	0.535452457
**Proteobacteria**	0.380860213	0.466759419	0.297148029	0.299609051
**Bacteria_ph**	0.101327325	0.09848449	0.261430321	0.308472218
**Cyanobacteria**	0.057117246	0.042546163	0.108038838	0.05544829
**Verrucomicrobia**	**2.088066581**	2.159629286	**0.07798574**	0.104098689
**Epsilonbacteraeota**	0.028560695	0.031495675	0.001783294	0.003987567
**Patescibacteria**	0.010080253	0.011656221	0.001685204	0.00376823
**Spirochaetes**	0.663707193	0.934527339	0	0
**WPS-2**	0.060361654	0.081889499	0	0

Table S4: The average proportions of Barbary macaque fecal microbiota at genus level between the TP and WF groups.

Genus	TPG		WFG		p-values	
	**Average proportions (%)**	**S.D (%)**	**Average proportions (%)**	**S.D (%)**	**p-values**	**corrected**
Ruminococcaceae_UCG-002	**8.22096752**	**7.0761071**	**28.0225812**	**16.9302915**	**0.04813761**	**0.67713577**
Ruminococcaceae_ge	**4.077813668**	**2.89077892**	**20.6580866**	**15.1114187**	**0.05747591**	**0.71337743**
Ruminococcus_2	**21.09481273**	**10.4449885**	**0.62848252**	**0.61090222**	**0.00708275**	**0.37361491**
Ruminococcaceae_UCG-005	**4.116826587**	**2.38856974**	**12.229966**	**1.54294056**	**0.00016015**	**0.03379235**
Faecalibacterium	**15.90466835**	**12.96292**	**0.53312671**	**0.39644314**	**0.04532514**	**0.73566188**
Christensenellaceae_R-7_group	**6.630294098**	**2.74685809**	**1.0837972**	**0.36402852**	**0.00602445**	**0.42371994**
Subdoligranulum	**6.460949457**	**4.68995541**	**1.38162776**	**1.34721996**	**0.06015603**	**0.70516236**
Ruminococcaceae_UCG-010	**0.875854029**	**1.38892077**	**7.31382**	**5.14795669**	**0.03729085**	**0.78683684**
Dorea	**4.72331511**	**2.3782348**	**0.26436299**	**0.32859089**	**0.00819821**	**0.34596426**
Lachnospiraceae_ge	**3.152472345**	**1.82190392**	**2.67551564**	**1.6315939**	**0.67215572**	**0.72359621**
Blautia	**2.796397509**	**1.66430774**	**0.82936693**	**1.26619486**	**0.06367894**	**0.70717136**
Ruminococcaceae_UCG-013	**0.453843344**	**0.2030036**	**3.06293237**	**2.6735486**	**0.080913**	**0.74228883**
Ruminococcaceae_NK4A214_group	**0.352747653**	**0.1322385**	**2.36610889**	**1.50649851**	**0.03032927**	**0.79993457**
Erysipelotrichaceae_UCG-003	**3.285285733**	**3.78399277**	**0.46114911**	**0.37676798**	**0.15653782**	**0.76812744**
Prevotella_9	**0.104123456**	**0.06725261**	**2.11391167**	**2.19176007**	**0.09560356**	**0.80689402**
Clostridiales_Family_XIII_AD3011_group	**1.134299192**	**0.77431504**	**0.67798938**	**0.36542613**	**0.27157572**	**0.68217235**
Ruminococcaceae_UCG-014	**1.677586276**	**1.43836958**	**0.8706717**	**1.09163328**	**0.34290509**	**0.75367682**
Akkermansia	**2.088066581**	**2.15962929**	**0.07798574**	**0.10409869**	**0.091942**	**0.80832339**
Clostridiales_Family_XIII_ge	**1.286141769**	**1.72363984**	**1.0472682**	**1.50005778**	**0.81997501**	**0.86942074**
Dialister	**0.402175796**	**0.54161521**	**0.78299369**	**0.92463679**	**0.44955517**	**0.54830139**
Lachnospiraceae_NK4A136_group	**0.444445032**	**0.31284128**	**0.04110999**	**0.03647437**	**0.0341909**	**0.80158658**
Marvinbryantia	**0.613767399**	**0.69400072**	**0.02128221**	**0.03437064**	**0.11459658**	**0.83378892**

Clostridiales_vadinBB60_group_ge	**0.01847603**	**0.02422983**	**0.40004541**	**0.36596554**	**0.06706978**	**0.67389156**
Mollicutes_RF39_ge	**0.308450654**	**0.18946336**	**0.46769848**	**0.65017413**	**0.61836646**	**0.68311687**
Rikenellaceae_ge	**0.041919881**	**0.05079602**	**0.50624908**	**0.59609794**	**0.14233517**	**0.77006974**
Coprococcus_3	**0.482516245**	**0.42086207**	**0.12965872**	**0.17677867**	**0.12937606**	**0.80289262**
Bacteroidales_ge	**0.082304824**	**0.12243411**	**0.42005867**	**0.41903662**	**0.13564606**	**0.81775195**
Muribaculaceae_ge	**0.391965603**	**0.5209604**	**0.12604965**	**0.07475135**	**0.30791563**	**0.70619781**
Solobacterium	**0.228419124**	**0.13393793**	**0.01858528**	**0.01682345**	**0.01686735**	**0.50843025**
Candidatus_Stoquefichus	**0.193312001**	**0.18772405**	**0.17741607**	**0.29684759**	**0.9217517**	**0.95807689**
Ruminococcus_1	**0.273517981**	**0.32043287**	**0.42919689**	**0.82389918**	**0.70637924**	**0.75657878**
Bacteria_ge	**0.101327325**	**0.09848449**	**0.26143032**	**0.30847222**	**0.3111969**	**0.70604888**
Ruminiclostridium_6	**0.065536105**	**0.08996252**	**0.18852079**	**0.11845272**	**0.09636694**	**0.7820548**
Gastranaerophilales_ge	**0.057117246**	**0.04254616**	**0.10803884**	**0.05544829**	**0.13637332**	**0.79929921**
Coprococcus_1	**0.365552337**	**0.4072913**	**0.05444758**	**0.05833399**	**0.14936475**	**0.78789906**
Rhodospirillales_ge	**0.01011003**	**0.01166408**	**0.15487377**	**0.16165206**	**0.10170855**	**0.79483345**
Coriobacteriales_ge	**0.065567743**	**0.09943914**	**0.37138423**	**0.44717835**	**0.19056543**	**0.62827039**
Lactobacillus	**0.201135341**	**0.23605452**	**0.02201995**	**0.03574008**	**0.15170099**	**0.78070509**
Christensenellaceae_ge	**0.077341493**	**0.02445028**	**0.04542072**	**0.04483099**	**0.20097713**	**0.63292799**
Ruminiclostridium_5	**0.099197271**	**0.03748149**	**0.01552093**	**0.01470373**	**0.00284992**	**0.30066673**
Fusicatenibacter	**0.179433876**	**0.20118691**	**0.06454182**	**0.0982924**	**0.2876482**	**0.7057415**
Rikenellaceae_RC9_gut_group	**0.067399539**	**0.05795919**	**0.5864638**	**1.19579124**	**0.37663267**	**0.47873189**
Prevotellaceae_ge	**0.035276514**	**0.02160297**	**0.42177236**	**0.81455924**	**0.33732598**	**0.74921875**
Escherichia-Shigella	**0.099144736**	**0.07468659**	**0.0072193**	**0.00827121**	**0.03997709**	**0.76683335**
Clostridiales_ge	**0.026850073**	**0.02439611**	**0.08617842**	**0.06679341**	**0.1089437**	**0.82096861**
Holdemanella	**0.132076582**	**0.21142416**	**0.0424996**	**0.03666657**	**0.39112846**	**0.49418027**
Ruminococcaceae_UCG-003	**0.088985924**	**0.09277447**	**0.03313923**	**0.02828822**	**0.24594366**	**0.64867639**
Ruminococcaceae_UCG-009	**0.003352276**	**0.00474084**	**0.16481511**	**0.23161045**	**0.17980416**	**0.61191415**
Agathobacter	**0.001677571**	**0.00375116**	**1.21860526**	**2.39617236**	**0.30760256**	**0.71323232**
Phascolarctobacterium	**3.521397617**	**7.70806465**	**0.0018754**	**0.00419352**	**0.35409001**	**0.77023702**

Alloscardovia	**0.060462834**	**0.03427715**	**0**	**0**	**0.01091291**	**0.38377073**
Burkholderiaceae_ge	**0**	**0**	**0.05661436**	**0.05418336**	**0.06667877**	**0.70346103**
Ruminiclostridium_9	**0.036893871**	**0.02823094**	**0.04993038**	**0.0573658**	**0.6617152**	**0.71600978**
Coprococcus_2	**0.224613721**	**0.34836854**	**0.01384807**	**0.01466189**	**0.23422572**	**0.65028457**
Succinivibrio	**0.224620318**	**0.31419098**	**0.03770354**	**0.02653142**	**0.24156125**	**0.64518259**
Clostridium_sensu_stricto_1	**0.005026138**	**0.00767579**	**0.10655054**	**0.13534949**	**0.15449892**	**0.77617314**
Lachnospiraceae_ND3007_group	**0.003351432**	**0.00473964**	**0.09042096**	**0.12254811**	**0.17308417**	**0.77703746**
Streptococcus	**0.038785695**	**0.03016069**	**0.01640609**	**0.02403699**	**0.22498987**	**0.6329715**
Coriobacteriales_ge_ge	**0.010113922**	**0.0082694**	**0.05716279**	**0.0882741**	**0.28782209**	**0.69805128**
Anaeroplasma	**0.010096477**	**0.01430234**	**0.05794367**	**0.07443636**	**0.21333647**	**0.60829724**
Anaerosporobacter	**0.04027653**	**0.03528731**	**0**	**0**	**0.05112766**	**0.67424604**
Alistipes	**0**	**0**	**0.05613423**	**0.06624919**	**0.11665951**	**0.79403731**
Lachnospiraceae_UCG-010	**0.015092407**	**0.02582476**	**0.02434147**	**0.01640404**	**0.51707497**	**0.59946603**
Ruminococcaceae_UCG-008	**0.025238426**	**0.02834956**	**1.64118005**	**3.65067231**	**0.36773963**	**0.47603105**
Butyricicoccus	**0.015117267**	**0.01265576**	**0.0266144**	**0.02775872**	**0.42728353**	**0.52723289**
Sarcina	**0.045562246**	**0.05133229**	**0.00524581**	**0.00785762**	**0.1404553**	**0.8009748**
Anaerostipes	**0.067310564**	**0.08909995**	**0.01561262**	**0.02003068**	**0.2565107**	**0.66819455**
Lachnospira	**0.020193524**	**0.02665597**	**0.06379742**	**0.0775523**	**0.27822522**	**0.6906532**
Oxalobacter	**0.015107235**	**0.0112791**	**0.01357515**	**0.02157247**	**0.89176685**	**0.93149904**
Olsenella	**0.075493185**	**0.10307429**	**0**	**0**	**0.16240405**	**0.76149453**
Prevotellaceae_UCG-001	**0.051959674**	**0.06264869**	**0**	**0**	**0.12282757**	**0.78535205**
Parabacteroides	**0.013412304**	**0.01382776**	**0.01393962**	**0.01640156**	**0.9572807**	**0.98051567**
Ralstonia	**0**	**0**	**0.01521364**	**0.01263568**	**0.043181**	**0.75926598**
Anaerofustis	**0.03357577**	**0.04672558**	**0.01186915**	**0.01476386**	**0.36024355**	**0.76011388**
Campylobacter	**0.028560695**	**0.03149567**	**0.00178329**	**0.00398757**	**0.11615555**	**0.81696072**
Defluviitaleaceae_UCG-011	**0.03018414**	**0.03842536**	**0.11551469**	**0.25354372**	**0.48890708**	**0.58282143**
Roseburia	**0.103953267**	**0.13661921**	**0.00514742**	**0.00515142**	**0.16684645**	**0.76531739**
Lachnospiraceae_UCG-001	**0.023495213**	**0.03320011**	**0.0471675**	**0.07606443**	**0.54436628**	**0.62424611**

Intestinibacter	**0.006698821**	**0.01497902**	**2.88385589**	**6.42490919**	**0.36262983**	**0.7575732**
Anaerovibrio	**0.011769538**	**0.00905917**	**0.00334504**	**0.00747974**	**0.14100078**	**0.78292539**
Cellulosilyticum	**0**	**0**	**0.0220531**	**0.02633366**	**0.12001306**	**0.79133613**
Rickettsiales_fa_ge	**0**	**0**	**0.00860324**	**0.00728312**	**0.04590031**	**0.69178323**
Treponema_2	**0.663707193**	**0.93452734**	**0**	**0**	**0.17313555**	**0.76107504**
WPS-2_ge	**0.060361654**	**0.0818895**	**0**	**0**	**0.16022083**	**0.7683317**
Bacteroides	**0.006700843**	**0.01110935**	**0.01066891**	**0.01267946**	**0.61032414**	**0.67778102**
Alloprevotella	**0.031859226**	**0.05820261**	**0.04214533**	**0.08073989**	**0.82236357**	**0.86759356**
Ruminococcaceae_V9D2013_group	**0.001674705**	**0.00374475**	**0.01039956**	**0.01219295**	**0.177536**	**0.61409994**
Saccharimonadaceae_ge	**0.010080253**	**0.01165622**	**0.0016852**	**0.00376823**	**0.17604243**	**0.61908254**
Fastidiosipila	**0.023552091**	**0.03630382**	**0**	**0**	**0.20657661**	**0.61391077**
Rhodospirillales_fa_ge	**0.006709164**	**0.00748967**	**0.00334504**	**0.00747974**	**0.49353091**	**0.58502821**
Atopobiaceae_ge	**0.00505053**	**0.00505073**	**0**	**0**	**0.07559506**	**0.72502534**
Peptococcaceae_ge	**0.010070534**	**0.01423468**	**0**	**0**	**0.17450775**	**0.75145174**
Lachnospiraceae_FCS020_group	**0.39212006**	**0.58223454**	**0.00523911**	**0.00810302**	**0.19748218**	**0.63134455**
Psychrobacter	**0.02516873**	**0.04744899**	**0**	**0**	**0.28887043**	**0.6926325**
Erysipelotrichaceae_UCG-004	**0**	**0**	**0.06136537**	**0.09604684**	**0.21247827**	**0.62267937**
GCA-900066225	**0.00336025**	**0.00475215**	**0.00514136**	**0.00514564**	**0.58224598**	**0.65002064**
